# Psychometric properties of cognitive screening for patients with cerebrovascular diseases A systematic review

**DOI:** 10.1590/1980-57642018dn13-010004

**Published:** 2019

**Authors:** Jaqueline de Carvalho Rodrigues, Natália Becker, Carolina Luísa Beckenkamp, Camila Schorr Miná, Jerusa Fumagalli de Salles, Denise Ruschel Bandeira

**Affiliations:** 1 Psicóloga, Mestre e Doutora em Psicologia, Universidade Federal do Rio Grande do Sul, Porto Alegre, RS, Brazil.; 2 Psicóloga, Mestre e Doutoranda em Psicologia, Universidade Federal do Rio Grande do Sul, Porto Alegre, RS, Brazil.; 3 Mestranda em Neurociências, Rheinische Friedrich-Wilhelms-Universität Bonn, Bonn, Germany.; 4 Psicóloga, Mestranda em Neurociências, Universidade Federal do Rio Grande do Sul, Porto Alegre, RS, Brazil.; 5 Professora Associada do Instituto de Psicologia, Universidade Federal do Rio Grande do Sul, Porto Alegre, RS, Brazil.; 6 Professora Titular do Instituto de Psicologia, Universidade Federal do Rio Grande do Sul, Porto Alegre, RS, Brazil.

**Keywords:** neuropsychological assessment, stroke, vascular cognitive impairment, validity, reliability, avaliação neuropsicológica, acidente vascular cerebral, comprometimento cognitivo vascular, validade, fidedignidade

## Abstract

Screening instruments are ideal for acute clinical settings because they are easy to apply, fast, inexpensive and sensitive for specific samples. However, there is a need to verify the psychometric properties of screening in stroke patients.

**Objective:**

This study investigated the psychometric properties (methodological procedures) of cognitive screening for patients with cerebrovascular diseases.

**Methods:**

A systematic review of papers published on PsycINFO, Web of Knowledge, PubMed and Science Direct (2005 to 2016) was performed.

**Results:**

A total of 55 articles remained after applying exclusion criteria. The samples ranged from 20 to 657 patients. Most articles evaluated elderly individuals with four to 13 years of education who had experienced ischemic or hemorrhagic stroke. There was a tendency to find evidence of validity for criteria and to analyze the sensitivity/specificity of the instruments. Although the studies frequently used the Mini-Mental State Examination (MMSE) and the Montreal Cognitive Assessment (MoCA) to seek evidence of validity and reliability, the use of these instruments among stroke patients has been criticized due to their psychometric properties and the neuropsychological functions evaluated.

**Conclusion:**

Although there is no gold standard screen for assessing adults post-stroke, instruments devised specifically for this population have shown promise. This review helps both researchers and clinicians to select the most appropriate screen for identifying cognitive impairment in adults post-stroke.

## RESUMO.

Instrumentos de triagem são ideias no contexto clínico hospitalar, uma vez que são fáceis de administrar, rápidos, tem baixo custo e são sensíveis para amostras específicas. Portanto, há a necessidade de se verificar as propriedades psicométricas de instrumentos de triagem para pacientes pós acidente vascular cerebral.

**Objetivo:**

Este estudo investigou as propriedades psicométricas (procedimentos metodológicos) de triagens cognitivas para pacientes com doenças cerebrovasculares.

**Métodos:**

Foi realizada uma revisão sistemática de artigos publicados em PsycINFO, Web of Knowledge, PubMed e Science Direct (2005 a 2016). Apenas 55 artigos permaneceram após a aplicação dos critérios de exclusão.

**Resultados:**

As amostras variaram de 20 a 657 pacientes; a maioria dos artigos avaliou indivíduos idosos, com quatro a 13 anos de educação, que sofreram AVC isquêmico e hemorrágico. Houve uma tendência para encontrar evidências de validade relacionadas ao critério e analisar a sensibilidade/especificidade dos instrumentos. Embora os estudos frequentemente apliquem o Mini Exame do Estado Mental (MMSE) e o Montreal Cognitive Assessment (MoCA) para buscar evidências de validade e de fidedignidade, o uso desses instrumentos em pacientes com AVC é criticado por razões relacionadas às suas propriedades psicométricas e funções neuropsicológicas avaliadas.

**Conclusão:**

Embora não exista uma triagem padrão-ouro para avaliação de adultos pós-AVC, os instrumentos construídos especificamente para esta população mostraram-se promissores. Este estudo de revisão contribui tanto aos pesquisadores quanto aos clínicos que desejam selecionar o rastreio mais apropriado para identificar comprometimento cognitivo em adultos pós-AVC.

Cognitive impairment is a common consequence following stroke, occurring in approximately 45% to 83% of subjects depending on the follow-up time, neurological characteristics and instruments used.^1^^,^ 2 Notably, cognitive impairment is observed in more than 50% of patients six months after stroke.^2^^,^ 3 These patients can develop vascular dementia, which affects both functional independence and quality of life.^4^^-^ 6

The most common deficits in vascular cognitive impairment include reduced processing speed, executive dysfunction, hemineglect, inattention, aphasia, apraxia and amnesia.^3^^,^ 5 ^,^ 7 There is no consensus on which tests should be used to evaluate performance on these functions in post-stroke patients.^8^ The selection of tools usually depends on an instrument’s availability and on the neuropsychologist’s preference and familiarity with the tasks.^9^

Using an extensive battery of neuropsychological assessment is impractical in many clinical settings, where evaluation with simpler cognitive screening instruments is required.^9^ Screening instruments are therefore ideal for an acute clinical setting because they are easy to apply, fast, inexpensive and sensitive for specific samples.^10^^,^ 11 Ideally, neuropsychologists should be aware of whether the selected screen has adequate psychometric properties for stroke populations in their countries. However, most neuropsychologists have based their diagnosis on instruments psychometrically tested in patients with nonvascular cognitive impairment.^6^^,^ 12 ^-^ 14 The Neuropsychological Working Group of the National Institute of Neurological Disorders and Stroke (NINDS) and the Canadian Stroke Network (CSN) have recommended three protocols (60, 30, and 5 minute protocols) for assessing vascular cognitive impairment,^15^ and their psychometric properties have been tested in many studies.^16^^-^ 20

Regarding the instruments’ psychometric properties, neuropsychological tests should exhibit evidence of specific forms of validity: evidence based on test content, evidence based on response processes, evidence regarding internal structure (dimensionality and relationships between scores of the same test), evidence regarding relationships with conceptually related constructs (convergent and discriminant evidence), evidence regarding relationships with criteria (contrasting groups, effect size, concurrent and predictive validity), and evidence based on the consequences of testing. In addition, it is important for the tests to demonstrate reliability in the form of temporal stability and internal consistency.^21^

Furthermore, the instrument should be constructed in a manner that aims to determine cognitive deficits in a specific population. Thus, we recognize the need to verify the psychometric properties of screening in stroke patients. In this context, the present systematic review aims to identify the cognitive screening with adequate psychometric properties for use in stroke patient samples. The specific aims of this review study were: (a) to analyze the quality of the methodological information reported (sample size, age and education of participants, neurological data such as cerebrovascular disease and time post-stroke); and (b) to identify cognitive screening that have adequate validity and reliability evidence. This systematic review reports the methodological limitations of psychometric studies of adults post-stroke and investigates which screening are most adequate for identifying cognitive deficits in these patients. This review article can be distinguished from other studies in this field that tend to discuss only the sensitivity and specificity of screening instruments^22^^,^ 23 or fail to examine the psychometric properties of the tests in stroke patient samples.^8^

## METHODS

We performed a systematic review of papers published from January 2005 to December 2016 on the following databases: PsycINFO (refined by the terms in the abstract), Web of Knowledge (refined by the terms in the subject of the article), PubMed (refined to include the terms in the title and abstract), and Science Direct (refined by the terms in the abstract, title, or keywords). The refinements varied because the databases use different advanced search tools. The following combinations of keywords were applied: “stroke”, “cerebrovascular accident”, “vascular cognitive impairment”, and “cerebrovascular disease” versus “neuropsychological assessment”, “neuropsychological evaluation”, “cognitive screening”, “neuropsychological screening”, “cognitive assessment”, and “cognitive evaluation”. These keywords were selected from the most commonly used terms in the health databases to include all articles that reported neuropsychological evaluations in stroke patients.

After excluding repeated articles, the remaining articles were divided and analyzed by two judges. Four judges selected only empirical studies in English, Portuguese, French or Spanish that assessed adults with cerebrovascular disease using cognitive screening. If the two judges disagreed on the selection of a particular study, a third judge was recruited. The judges had experience in neuropsychological assessment post-stroke and knowledge about the instruments used.

Many studies have evaluated neuropsychological deficits in stroke patients with cognitive screening tests, but failed to explicitly report that their analysis was psychometric. In these situations, we assumed that these articles claimed to analyze validity evidence based on relations to other variables.^21^

## RESULTS

**Figure 1:**
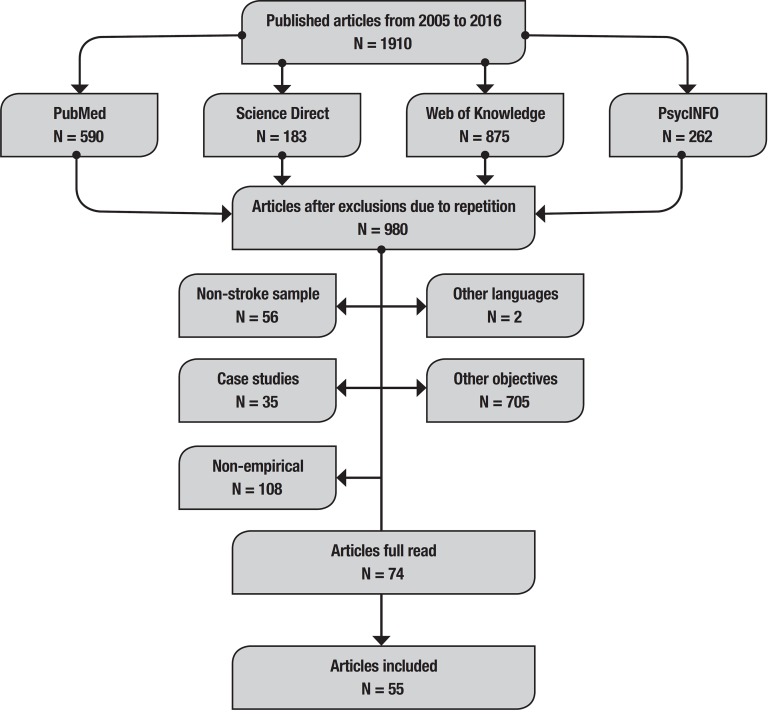
Studies selection process flowchart.

After performing searches and excluding repeated articles, 74 studies that evaluated neuropsychological functions in stroke groups using screening instruments were selected. These articles were read in full, with a focus on the methods and results sections. Subsequently, 19 non-psychometric studies were excluded (Figure 1). The results and discussion will be presented in two sections: (1) characteristics of the samples; and (2) psychometric properties of the cognitive screening.

### Characteristics of the samples

In our review, the samples ranged from 20 to 657 stroke patients, but only one study calculated the sample size.^24^ Most of the articles evaluated individuals between 50 and 80 years old (Table 1), and only two studies included younger samples (i.e., patients under 30 years of age).^25^^,^ 26 The majority of the studies evaluated patients with four to 13 years of education (9 years on average). However, 27.27% of the articles did not specify the educational levels of the participants (Table 1). With respect to cerebrovascular disease, 30.90% of studies evaluated ischemic and hemorrhagic stroke samples, 21.81% evaluated transient ischemic attack (TIA) and stroke patients, 10.90% ischemic stroke only, 9.09% cerebral small vessel diseases, 7.27% hemorrhagic stroke, 3.63% vascular dementia and 16.36% did not report this information (Table 1). The time between post-stroke onset and neuropsychological assessment varied. A total of 50.90% of studies assessed patients at 3 months post-stroke, 25.45% included patients who were assessed from 3 to 12 months post-stroke and 14.54% assessed patients more than 12 months post-stroke. A total of 9.09% of the articles did not report time post-stroke (Table 1).

**Table 1:** Cognitive screening and characteristics of the samples.

Cognitive screening	N (CVD)	Age (M±SD or range)	Years of education (M±SD, % or range)	Time post-stroke
Addenbrooke's Cognitive Examination-Revised (ACE-R)^13^	101 (NI)	67.0-82.5	9.0-11.0	8-48 days
Barrow Neurological Institute Screen for Higher Cerebral Functions (BNIS)^27^	54 (I, H, SH)	53.8±12.3	33.3% high education	15.0±12.8 weeks
	295 (I)	43.4-66	92 < 9; 203 > 9	7 years
Birmingham Cognitive Screen (BCoS - Cantonese version)^29^	98 (I)	>50 years	NI	2 weeks
Birmingham Cognitive Screen (BCoS)^30^	657 (I and H)	69.31±14.34, 71.38±12.60	11.52±2.76, 11.19±2.76	26.65±22.36, 20.44±17.29
Brief Memory and Executive Test (BMET)^31^	196 (SVD)	63.5±9.9	13.7±3.8	20.5±32.3 months
Brief Neuropsychological Screening (BNS)^32^	134 (NI)	69.7±12.9	8.3±3.4	<1 month
Clock Drawing Test (CDT)^7^	49 (I, IH, SH)	62±53.5	NI	38±17 days
CDT^33^	187 (I and H)	49 - 80	0 - 29	3 - 25 months
Cog-4^34^	92 (I and TIA)	63-83	NI	1-4 days
Cognistat^7^	49 (I, IH, SH)	62±53.5	NI	38±17 days
Cognitive Assessment Scale for Stroke Patients (CASP)^35^	44 (I and H)	64±15	NI	42±22 days
CASP^36^	50 (I and H)	63±14	NI	40±17 days
Functional Independence Measure (FIM cognitive)^37^	169 (I and H)	56.2±11.3	61.2% intermediate	<12 months
Middlesex Elderly Assessment of Mental State (MEAMS)^38^	30 (NI)	75.8±7.94	NI	20.73±24.37 days
Mild Vascular Cognitive Impairment Assessment tool for Stroke (MVCI)^39^	60 (I and H)	64.07±13.46	NI	NI
Mini-Mental State Examination (MMSE)^6^	34 (I and H)	64.7±11.5	1 - 7	6.5±2.9 days
MMSE^37^	169 (I and H)	56.2±11.3	61.2% intermediate	<12 months
MMSE^40^	493 (TIA, I and H)	69.9±12.4	NI	6 months or 5 years
MMSE^41^	80 (MI, SVD)	65.85±6.94	10.00±3.35	3 months
MMSE^42^	239 (TIA and I)	60.2±11.8	7.5±4.1	3-6 months
MMSE^43^	327 (TIA and I)	59.8±11.6	7.7±4.3	3-6 months
MMSE^24^	60 (I and IH)	72.1±13.9	10.5±3.9	98.3±12.0 days
MMSE^44^	388 (I and TIA)	59.8±11.6	7.7±4.3	0-14 days, 3-6 months
MMSE^45^	138 (SIVD)	50-85	> 6	> 3 months
MMSE^46^	105 (I)	68.61±10.35	8.90±4.24	< 2 weeks
MMSE^16^	83 (I and H)	66.6±9.7	9.2±4.8	9.0±5.4 months
Montreal Cognitive Assessment (MoCA)^43^	327 (TIA and I)	59.8±11.6	7.7±4.3	3-6 months
MoCA^47^	29 (I, H, TIA)	68±12	NI	2.5±1.4 days
MoCA^48^	95 (I and H)	68.2±13.7	79% primary	6.6±3.5
MoCA^20^	34 (VD)	73.21±7.85	4.97±2.74	NI
MoCA^42^	239 (TIA and I)	60.2±11.8	7.5±4.1	3-6 months
MoCA^14^	99 (TIA and stroke)	73.4±11.6	63% < 12	1 year
MoCA^24^	60 (I and IH)	72.1±13.9	10.5±3.9	98.3±12.0 days
MoCA^49^	80 (I and H)	68.2±14.6	9.2±4.4	5-9 days, 8.4±2.2 months
MoCA^44^	388 (I and TIA)	59.8±11.6	7.7±4.3	0-14 days, 3-6 months
MoCA^12^	136 (I and H)	64.3±14.3	NI	11.1±14.5 days
MoCA^25^	108 (aSAH)	21-75	NI	2-4 weeks, 1 year
MoCA^26^	194 (aSAH)	21-75	NI	1 year
MoCA^45^	138 (SIVD)	50-85	> 6	> 3 months
MoCA^50^	211 (I and H)	70.6±1.8	23/98/42 (low/medium/ high)	5.1±4.9 days
MoCA^18^	291	59.8±11.6	7.7±4.3	3-6 months, 1 year
MoCA^46^	105 (I)	68.61±10.35	8.90±4.24	< 2 weeks
miniMoCA^51^	72 (NI)	68.1±15	11.2±2	NI
MoCA (by telephone)^52^	91 (I, H, TIA)	72.9±11.6	63% <12	3.1±1.9 years
MoCA BM (Bahasa Malaysia version)^53^	40 (I and H)	57.2±10.3	8.5±3.9	164-581 days
MoCA Beijing version^54^	102 (I and TIA)	53.95±11.43	12/74/26 (low/medium/ high)	> 14 days
MoCA Beijing version (by telephone)^19^	89 (NI)	62.9±8.6	9.2±4.2	> 3 months
MoCA (Changsha)^55^	338 (I and VD)	> 40 years	> 2	NI
MoCA (Chinese - Cantonese)^56^	70 (VD)	70.1±7.88	8.84±3.20	3 months
MoCA (Chinese - Cantonese)^57^	a) 74, b) 80 (aSAH)	a) 49-66, b) 47-61	NI	a) 2-7 weeks, b) 1 year
MoCA (Chinese)^58^	206 (I)	68.14±10.64 (NCI), 69.24± 11.45 (VCIND)	9.73±5.35 (NCI), 8.65±5.51(VCIND)	NI
Hong Kong MoCA (HK-MoCA)^59^	90 (aSAH)	54.0±11.0	NI	3 months
HK-MoCA^57^	50 (I)	68.8±9.2	5.9±4.1	6-18 months
HK-MoCA (by telephone)^60^	104 (I, H, and TIA)	68.9±10.1, 70.8±9.2	6.3±4.4, 6.0±4.5	39.4±7.6 months
HK-MoCA^61^	40 (SMD)	70.08±8.5	5.98±4.5	< 3 months
MoCA (Singaporean)^62^	100 (TIA, I and H)	61.2±11.3	52% ≤ primary	4.2±2.4 days
Northwick Park Examination of Cognition (NPEC)^63^	166 (NI)	69.2±14.1	NI	5.6±7.9 days
Oxford Cognitive Screen (OCS)^9^	208 (NI)	71.1±14.5	11.5±2.7	6.6±4.69 days
OCS^64^	200 (I and H)	70.5±14.7	NI	6.1±4.4 days
Repeatable Battery for Assessment of Neuropsychological Status (RBANS)^65^	a) 158, b) 36 (NI)	a) 64.27±14.45, b) 63.21±16.19	a) 34% < 12 b) 29% < 12	a) 20±19.4 days, b) 12 months
Rotterdam CAMCOG (R-CAMCOG)^37^	169 (I and H)	56.2±11.3	61.2% intermediate	<12 months
Screening Instrument for Neuropsychological Impairments in Stroke (SINS)^7^	49 (I, IH, SH)	62±53.5	NI	38±17 days
Six-item Screener (SIS - by telephone)^19^	89 (NI)	62.9±8.6	9.2±4.2	> 3 months
Weigl's Colour-Form Sorting Test (WCFT)^66^	105 (NI)	73.4±10.7	9.9±1.7	NI
Zürich Maxi Mental Status Inventory (ZüMAX)^67^	33 (NI)	63.2±14.7	NI	49.4±79.8 days

N: number of participants; NI: not informed; CVD: cerebrovascular disease; MI: multiple infarcts; SVD: small vessel disease; TIA: transient ischemic attack; I: ischemic; H: hemorrhagic; IH: intracerebral hemorrhage; SH: subarachnoid hemorrhage; VD: vascular dementia; aSAH: aneurysmal subarachnoid hemorrhage; SIVD: subcortical ischemic vascular disease.

### Psychometric properties of the instruments

In the last few years, many studies have demonstrated the psychometric properties of the instruments according to the tripartite model of validity: content, criteria and construct. However, in our systematic review, we classified the evidence of validity and reliability in accordance with recently established definitions.^21^ Most of the studies determined validity based on relationships with criteria (60%), relationships with related constructs (22.50%), and content-oriented evidence (0.83%) (Table 2). Only 19 articles presented data on the reliability of the instruments, 10.83% of which discussed internal consistency reliability and 5% temporal stability reliability (Table 2).

**Table 2:** Cognitive screenings,procedures for determining validity/reliability.

Cognitive screening	Procedures for determining validity/reliability with appropriate values	Procedures for determining validity/reliability without appropriate values
Addenbrooke's Cognitive Examination-Revised (ACE-R)		SE/SP (relationships with criteria)^13^
Barrow Neurological Institute Screen for Higher Cerebral Functions (BNIS)	Concurrent validity (relation with criteria),^27^^,^ 28 SE (relation with criteria),^27^ convergent validity (relation with related constructs),^27^^,^ 28 correlation (internal consistence reliability),^27^^,^ 28 comparison between contrasting groups (relation with criteria).^28^	SP (relation with criteria)^27^
Birmingham Cognitive Screen (BCoS - Cantonese version)	Inter-rater (internal-consistency reliability),^29^ test-retest (temporal stability reliability),^29^ comparison between contrasting groups (relation with criteria),^29^ convergent validity (relation with related constructs).^29^	
Birmingham Cognitive Screen (BCoS)	Comparison between contrasting groups (relation with criteria),^30^ predictive validity (relation with criteria).^30^	
Brief Memory and Executive Test (BMET)	Inter-rater (internal consistency reliability),^31^ test-retest (temporal stability reliability),^31^ SE/SP (relation with criteria),^31^ comparison between contrasting groups (relation with criteria),^31^ response times (response processes).^31^	
Brief Neuropsychological Screening (BNS)	Comparison between contrasting groups (relation with criteria),^32^ predictive validity (relation with criteria),^32^ convergent validity (relationship with related constructs).^ 32^	
Clock Drawing Test (CDT)	SE/SP (relation with criteria)^33^	Convergent validity (relation with related constructs),^7^SE/SP (relation with criteria).^7^
Cog-4		Convergent validity (relation with related constructs),^34^ SE/SP (relation with criteria).^34^
Cognistat		Convergent validity (relation with related constructs),^7^ SE/SP (relation with criteria).^7^
Cognitive Assessment Scale for Stroke Patients (CASP)		Convergent validity (relation with related constructs).^35^^,^ 36
Functional Independence Measure (FIM cognitive)		Convergent validity (relationship with related constructs)^37^
Middlesex Elderly Assessment of Mental State (MEAMS)		SE/SP (relation with criteria)^38^
Mild Vascular Cognitive Impairment Assessment tool for Stroke (MVCI)	Judge analysis - face validity (content-oriented evidence),^39^ convergent validity (relation with related constructs),^39^ SE/SP (relation with criteria),^39^ correlation,inter-rater (internal consistency reliability).^39^	
Mini-Mental State Examination (MMSE)	Comparison between contrasting groups (relation with criteria),^16^^,^ 24 ^,^ 41 ^,^ 45 ^,^ 46 SE (relation with criteria),^42^SE/SP (relation with criteria),^43^^,^ 46 predictive validity (relation with criteria),^24^concurrent validity (relation with criteria),^45^convergent validity (relation with related constructs).^16^,^45^	SE/SP (relation with criteria),^6^ convergent validity (relation with related constructs),^37^^,^ 40 predictive validity (relation with criteria),^42^^,^ 44 SP(relation with criteria),^42^ SE (relation with criteria).^24^^,^ 45
Montreal Cognitive Assessment (MoCA)	Comparison between contrasting groups (relation with criteria), 20 ^,^ 24 ^,^ 45 ^,^ 46 ^,^ 49 ^,^ 53 ^,^ 58 ^,^ 61SE/SP (relation with criteria),^18^^,^ 43 ^,^ 45 ^,^ 46 ^,^ 54 ^,^ 56 ^,^ 57 ^,^ 58 ^,^ 59 ^,^ 61 SE (relation with criteria),^ 12^^,^ 24 ^,^ 48 ^,^ 49 ^,^ 62 SP(relation with criteria),^20^convergent validity (relation with related constructs),^20^^,^ 45 ^,^ 49 ^,^ 53 ^,^ 55 ^,^ 56 ^,^ 61 inter-rater (internal-consistency reliability),^20^^,^ 55 ^,^ 61 predictive validity (relation with criteria),^18^^,^ 24 ^,^ 26 ^,^ 42concurrent validity (relation with criteria),^45^^,^ 53 ^,^ 61correlation (internal consistence reliability),^55^test-retest (temporal stability reliability).^ 55^^,^ 61	Concurrent validity (relation with criteria),^12^^,^ 47 SP(relation with criteria),^12^^,^ 42 ^,^ 48 ^,^ 49 ^,^ 62 SE (relation with criteria),^20^predictive validity (relation with criteria),^25^^,^ 50convergent validity (relation with related constructs).^59^
miniMoCA	SE/SP (relation with criteria),^51^convergent validity (relation with related constructs).^ 51^	
MoCA (by telephone)	Comparison between contrasting groups (relation with criteria),^19^^,^ 60 SE/SP (relation with criteria),^19^^,^ 52 ^,^ 60 convergent validity (relation with related constructs),^60^ concurrent validity (relation with criteria),^60^test-retest (temporal stability reliability),^60^inter-rater (internal consistency reliability).^60^	
Northwick Park Examination of Cognition (NPEC)	Comparison between contrasting groups (relation with criteria),^63^SE/SP (relation with criteria).^63^	
Oxford Cognitive Screen (OCS)	Convergent and discriminant validity (relation with related constructs),^9^SE/SP (relation with criteria),^9^ test-retest (temporal stability reliability),^9^ concurrent validity (relation with criteria),^64^ comparison between contrasting groups (relation with criteria).^64^	
Repeatable Battery for Assessment of Neuropsychological Status (RBANS)	Convergent and discriminant validity (relation with related constructs),^65^ predictive validity (relation with criteria).^65^	
Rotterdam CAMCOG (R-CAMCOG)	Convergent validity (relation with related constructs)^37^	
Screening Instrument for Neuropsychological Impairments in Stroke (SINS)	Convergent validity (relation with related constructs)^7^	SE/SP (relation with criteria)^7^
Six-item Screener (SIS - by telephone)	Comparison between contrasting groups (relation with criteria)^19^	SE/SP (relation with criteria)^19^
Weigl's Colour-Form Sorting Test (WCFT)	Convergent validity (relation with related constructs),^66^ comparison between contrasting groups (relation with criteria).^66^	
Zürich Maxi Mental Status Inventory (ZüMAX)	Comparison between contrasting groups (relation with criteria),^67^ test-retest (temporal stability reliability).^67^	

SE: sensitivity, SP: specificity.

With respect to the procedures adopted to determine validity and reliability, many of the studies included a sensitivity and specificity analysis (28.33%), considered convergent validity (or relations to other tests) (21.66%), compared contrasting groups (14.16%), executed predictive validity (9.16%), performed inter-rater analysis (7.5%), considered concurrent validity (5.83%), considered test-retest reliability (4.16%), tested for correlation with other measures (3.33%) and identified effect size (2.5%). The alternate form, discriminant evidence, response times and an analytical judgment of the instrument (face validity) were investigated once for each (0.83% overall).

As can be observed in Table 2, the Montreal Cognitive Assessment (MoCA) followed by the Mini-Mental State Examination (MMSE) were the instruments most analyzed to find validity and reliability evidence (40.90% and 18.18%, respectively). Other instruments were investigated once or twice per instrument. The studies were classified according to appropriate or inappropriate values present in the Discussion section of the articles (Table 2).

We found 26 instruments whose psychometric properties had been investigated in stroke patients (Table 3). Table 3 shows the neuropsychological functions evaluated by the screening tools: episodic memory (80.77%), language (69.23%), orientation (65.38%), executive functions (61.54%), attention (53.84%), visuo-construction (38.5%), perception (34.61%), praxis (23.07%), abstraction (23.07%), arithmetic (19.23%) and processing speed (3.84%).

**Table 3:** Neuropsychological screening and functions evaluated.

Screening	Episodic memory	Language	Orientation	Executive functions	Attention	Visuo- construction	Perception	Praxis	Abstraction	Arithmetic	Processing speed
Addenbrooke's Cognitive Examination-Revised (ACE-R)	X		X	X	X	X					
Barrow Neurological Institute Screen for Higher Cerebral Functions (BNIS)	X	X	X		X		X				
Birmingham Cognitive Screen (BCoS)	X	X		X	X			X		X	
Brief Memory and Executive Test (BMET)	X		X	X							X
Brief Neuropsychological Screening (BNS)	X	X			X		X	X	X	X	
Clock Drawing Test (CDT)						X	X				
Cog-4		X	X	X	X						
Cognistat	X	X	X	X	X	X				X	
Cognitive Assessment Scale for Stroke Patients (CASP)	X	X	X	X		X		X			
Functional Independence Measure (FIM Cognitive)	X	X		X							
Middlesex Elderly Assessment of Mental State (MEAMS)	X	X	X	X			X				
Mild Vascular Cognitive Impairment Assessment tool for Stroke (MVCI)	X	X	X	X	X		X		X		
Mini-Mental State Examination (MMSE)	X	X	X		X	X				X	
MiniMoCA - Montreal Cognitive Assessment(miniMoCA)	X			X		X			X		
Montreal Cognitive Assessment (MoCA)	X	X	X	X	X						
MoCA (by telephone)	X		X	X	X						
MoCA (Hong Kong)		X	X	X	X						
MoCA (Singaporean)	X	X	X	X	X	X			X		
Northwick Park Examination of Cognition (NPEC)	X	X	X	X			X				
Oxford Cognitive Screening (OCS)	X	X	X		X		X	X		X	
Repeatable Battery for Assessment of Neuropsychological Status (RBANS)	X	X			X	X					
Rotterdam CAMCOG (R-CAMCOG)	X		X				X		X		
Screening Instrument for Neuropsychological Impairments in Stroke (SINS)		X				X		X			
Six-item Screener (SIS)	X		X								
Weigl's Colour-Form Sorting Test (WCFT)									X		
Zürich Maxi Mental Status Inventory (ZüMAX)	X	X		X		X	X	X			

## DISCUSSION

### Characteristics of the samples

Regarding sample size, we identified wide variability in the number of participants, and only one study presented a sample calculation. Calculating the sample size in psychometric studies is recommended both to avoid finding differences between groups by chance and to increase the likelihood of detecting true, clinically significant differences.^68^ Therefore, the results of many papers should be interpreted with caution because they do not use representative samples of stroke patients.

It is essential to ensure the sample’s representativeness by providing a detailed description of its sociodemographic and developmental characteristics in empirical studies.^21^ Most of the investigations involved elderly stroke patients (>60 years), and the psychometric properties of the screening are shown only for this age group. An increasing number of young people affected by this injury exhibit cognitive impairment, which is present in approximately 20% to 30% of young stroke patients.^3^ Age influences patient performance on cognitive tasks.^27^^,^ 28 ^,^ 60 Therefore, it is important to verify whether validity and reliability evidence vary according to this variable for each test.

Educational background may influence both patient performance and test sensitivity/specificity.^1^^,^ 9 However, several studies included in this review did not discuss the education of participants and did not control for this variable, which is a limitation.^7^^,^ 12 ^,^ 17 ^,^ 25 ^,^ 29,35 ^,^ 36 ^,^ 38 ^,^ 39 ^,^ 40 ^,^ 47 ^,^ 59 ^,^ 63 ^,^ 64 ^,^ 67 ^,^ 69 Adults with high educational levels usually have better performance on neuropsychological assessments, and the cut-off points of tests should take this into account.^27^^,^ 28 ^,^ 45 ^,^ 58 ^,^ 60 ^,^ 61 Years of education should always be considered in empirical studies in neuropsychology.

In relation to neurological variables, many studies did not report the cerebrovascular disease of the participants (16.36%). Patients present vascular cognitive impairment regardless of stroke type,^49^ although there are differences in the neuropsychological performance of patients with vascular dementia (VD), subcortical ischemic vascular disease (SIVD) and mild cognitive impairment (MCI).^45^ Therefore, future studies could provide validity evidence and cut-off points for the screening according to cerebrovascular disease (when differences are found between groups). This would enable clinicians to know when significant deficits are present in each case.

Lastly, the time post-stroke is important to note in empirical studies because instruments have shown different cut-off points and because patients recover some neuropsychological functions approximately six months post-stroke.^9^^,^ 18 ^,^ 30 ^,^ 31 ^,^ 49 ^,^ 55 ^,^ 61 Neuropsychological assessment is indicated after acute stroke. The early recognition of cognitive deficits leads to improved interventions and thus prognosis.^7^

### Psychometric properties of the instruments

Most instruments have shown validity regarding relationships with criteria, and the studies typically used age, education, stroke type and neuropsychological performance differentiations between clinical and control groups as criteria. This evidence is important in determining whether a neuropsychological instrument can predict either the performance of a specific group of individuals or whether there will be differences in the scores of contrasting groups.^21^ However, a stroke may produce different behavioral changes in individuals, thus complicating the definition of a criterion group. Although heterogeneity of performance is important for identifying the test’s psychometric properties, heterogeneity of lesions can limit the interpretation of the results for all types of cerebrovascular diseases.

Evidence based on relationships with related constructs was also one the most common forms of validity evidence found by the screening. Correlation with other tests and measures (related constructs) is important for proving that an instrument assesses the intended cognitive domains.^21^ In general, cognitive screening have been related with other instruments in that they evaluate similar neuropsychological functions. However, the strength of the correlation between instruments varied widely due to the different characteristics of the tests. For example, the CASP showed weak correlation with MoCA and the MMSE likely because it has visual items that can be administered to patients with severe expressive aphasia, while the other screening are language-dependent.^35^ Therefore, interpreting evidence of validity based on conceptually related constructs should be carried out with caution.

Other psychometric procedures, such as seeking content validity, may not have been found frequently because most of the screening instruments were not specifically devised for stroke samples. Further evidence of validity should be found in the manuals of the tests published in each country. Our study is limited by a failure to describe these data.

Most of the studies analyzed only the validity - not the reliability - of the instruments. We suggest that psychometric studies include analyses of reliability to enlarge their evidence and avoid measurement errors. For example, some studies with test-retest reliability (temporal stability reliability) demonstrated that patients have better performance on the reevaluation.^9^^,^ 18 ^,^ 33 ^,^ 34 Other studies show temporal score stability.^29^^,^ 31 ^,^ 55 ^,^ 60 ^,^ 61 ^,^ 67 Several studies did not specify the time of cognitive evaluation.^20^^,^ 39 ^,^ 51 ^,^ 55 ^,^ 58 ^,^ 66 Therefore, future studies should clarify the timing of the evaluation and show evidence in accordance with this variable.

Regarding psychometric property procedures, sensitivity and specificity analysis were the most commonly used in the studies. The sensitivity of a test relates to the percentage of individuals with deficits that the instrument is able to identify (true positive rate). In contrast, the specificity indicates the test’s ability to detect healthy people for the neuropsychological functions measured (true negative rate). According to Blake et al.,^70^ a cognitive screening instrument should have values superior to 80% and 60% for good sensitivity and acceptable specificity, respectively. However, many screening instruments did not reach these values.^6^^,^ 7 ^,^ 14 ^,^ 19 ^,^ 20 ^,^ 24 ^,^ 27 ^,^ 38 ^,^ 42 ^-^ 45 ^,^ 48 ^,^ 61 ^,^ 62 ^,^ 66 ^,^ 69 Therefore, items need to be better studied and replaced to improve the quality of the instruments.

Notably, convergent validity and comparisons between contrasting groups were frequently executed. These procedures are important to seek evidence of validity based on relationship with criteria, as previously discussed. Differences between contrasting groups with various degrees of severity of vascular cognitive impairment were highlighted in many studies.^14^^,^ 19 ^,^ 24 ^,^ 31 ^,^ 41 ^,^ 45 ^,^ 46 ^,^ 49 ^,^ 58 ^,^ 60 ^,^ 62 ^,^ 66 However, studies need to improve the control of variables such as sociodemographic (age and education) and neurological data (cerebrovascular disease) that influence patient cognitive performance.^4^^,^ 46

In this review article, most cognitive screening used in stroke samples were originally developed to evaluate MCI and Alzheimer dementia patients, such as the MMSE, MoCA, WCFT, R-CAMCOG, ACE and CDT. However, there is no theoretical basis to justify the use of such screening, and they do not contain specific tasks for stroke patients. The application of neuropsychological instruments with a theoretical base is important both to justify patient deficits and plan their rehabilitation.

The NINDS and the CSN recommended the use of the MoCA to evaluate vascular cognitive impairment as an alternative to the MMSE.^15^ These instruments are correlated.^20^^,^ 45 ^,^ 55 ^,^ 56 ^,^ 61 However, one advantage of the MoCA is that the ceiling effects were substantially less evident than for the MMSE in stroke patients.^14^^,^ 24 ^,^ 26 ^,^ 62 Although both instruments are commonly investigated, the applicability to stroke samples has been discussed.^12^^,^ 14 ^,^ 24 ^,^ 25 ^,^ 44 ^,^ 47 ^-^ 50 ^,^ 53 ^,^ 59 ^,^ 62

Some studies support the high sensitivity of the MoCA^18^^,^ 46 ^,^ 51 ^,^ 54, but reveal its low specificity.^12^^,^ 14 ^,^ 24 ^,^ 42 ^,^ 48 ^,^ 49 ^,^ 52 ^,^ 57 ^,^ 62 Chan et al.^12^ found that 77% of patients were classified as cognitively intact on the MoCA but were impaired for one or more cognitive domains on a neuropsychological assessment (intellectual functioning, processing speed, and visual memory) not evaluated by the screen. The MoCA also failed to identify patients without problems in daily life functioning after mild stroke^26^^,^ 44 ^,^ 47 ^,^ 59 and discharge destination;^50^ however, a relationship between the MoCA and functional measures was found post-stroke.^17^^,^ 26

The MoCA has demonstrated wide validity and reliability in several languages. However, researchers should exercise caution with MoCA cut-off points in each country because this test is influenced by educational level,^45^^,^ 58 ^,^ 60 ^,^ 61 age,^60^ cerebrovascular disease^14^^,^ 19 ^,^ 24 ^,^ 45 ^,^ 58 ^,^ 60 and time post-stroke.^49^^,^ 57 Additionally, deficits in language (comprehension and expression) and perception (hemineglect), which are common post-stroke, may negatively affect the performance of participants on MoCA tasks.

A limitation of the studies on the MoCA is that the cut-off point for elderly samples without vascular disease, as well as cut-off points from different countries generally, to classify cognitive impairment patients,^49^ underestimate the possible deficits post-stroke. It is also important to show cut-off points by subtest (cognitive function), which could contribute to understanding the impact of brain injury on specific skills.^62^

The MMSE is more specific than the MoCA,^24^^,^ 46 but is less sensitive for stroke patients.^6^^,^ 43 ^,^ 45 This instrument can show differences between clinical and control groups^16^^,^ 17 ^,^ 41 and between various cerebrovascular diseases,^16^^,^ 24 ^,^ 46 but underestimates cognitive impairment post-stroke.^40^ However, the MMSE has shown low prediction ability for functional outcomes.^44^

According to Pendlebury et al.,^14^ the MMSE showed a ceiling effect in many subtests (naming, registration, reading and writing reaching near maximal scores) in amnestic, TIA and stroke groups. Moreover, the MMSE is insensitive for evaluating abstract reasoning, executive functioning, and visual perception/construction deficits that are present in subcortical lacunar strokes.^6^ Compared to a detailed neuropsychological battery of tests, the MMSE did not present adequate levels of sensitivity and specificity.^13^ However, refining cut-off scores by age and education can both improve the sensitivity of the MMSE (at the cost of specificity).^48^

Studies that performed sensitivity/specificity analyses with the MMSE showed that these values were no higher than 80%.^42^^,^ 43 As indicated by Stolwyk et al.,^22^ these scores suggest that 20% of patients with vascular cognitive impairments are not identified, which is unacceptable in clinical practice. Therefore, the MMSE is not recommended^6^^,^ 37 because it does not exhibit adequate psychometric properties for stroke patients.

Other cognitive screening instruments developed for MCI and dementia have been tested in stroke samples, but none are specific for this population (CDT, WCFT, ACE, ACE-R, R-CAMCOG, MEAMS, Cog-4, SIS, SINS and RBANS). The psychometric properties of these instruments are weak and insufficient in clinical practice. The BNIS,^28^ ZüMAX^67^ and Cognistat^7^ are cognitive screening developed for acquired brain lesions in general. The BNIS shows adequate psychometric properties,^28^ but does not measure neuropsychological functions usually impairment post-stroke. In contrast, the ZüMAX and Cognistat present little evidence of validity in small stroke samples. Psychometric studies with these tests require further evidence of validity and reliability and should determine the optimal cut-off level for stroke patients.

This review study found only seven cognitive screening that are specifically designed to evaluate stroke patients: the BCoS, OCS, BNS, CASP, MVCI, BMET and NPEC. The BCoS assesses attention, executive function, language, memory, numeric abilities and praxis and exhibits wide validity and reliability.^29^^,^ 30 However, the BCoS has evidence only in the country in which it was developed, as well as for a Cantonese version. Therefore, researchers from other countries (with different cultures and languages) should test it in their regions before applying it. The OCS is based on the BCoS and avoids the confounding effects of aphasia and neglect that are frequent in stroke patients.^9^^,^ 64 Demeyere et al.^64^ showed higher sensitivity for the OCS than the MoCA in detecting cognitive impairments in stroke patients (88% vs. 79%). Future studies could build on the evidence of validity and reliability of this instrument, as well as provide broad normative data for other countries.

The format of the CASP appears better suited than the MMSE or MoCA for use in stroke patients with severe neurovisual disorders^36^ and aphasia because it can be administered without using language.^35^ However, its psychometric properties have yet to be studied.^36^ The BNS^32^ and NPEC^63^ discriminated acute stroke patients with cognitive impairments from those without cognitive problems and can be used to determine different cognitive profiles according to the location of the lesion. The MVCI exhibits good validity and reliability, and the overall probability of correctly discriminating vascular cognitive impairment was 90.0%.^39^ The BMET correctly identified 78% of patients with cognitive impairment,^31^ but was tested only in cerebral small vessel disease patients and shows modest sensitivity.

Impairments in reasoning and executive functioning are the most frequent cognitive deficits in the early phase post-stroke.^6^ Executive functions, attention and processing speed are also the most impaired functions in long-term stroke patients.^5^ However, most neuropsychological screening do not include tasks that evaluate these functions because they are not developed for stroke patients, which justifies the construction of specific instruments. Moreover, the majority of the studies may underestimate patient deficits.

In summary, the psychometric properties of neuropsychological screening for stroke patients have been explored by initial analyses that did not use representative samples. Although the studies most frequently used the MMSE and the MoCA to find evidence of validity and reliability, the use of these instruments in stroke patients has been criticized due to their psychometric properties and the neuropsychological functions evaluated. Therefore, more studies involving specific instruments for stroke patients are necessary to confirm the validity and reliability of the cognitive screening.

## References

[1] Jokinen-Salmela H, Melkas S, Ylikoski R, Pohjasvaara T, Kaste M, Erkinjuntti T (2015). Post-stroke cognitive impairment is common even after successful clinical recovery. Eur J Neurol.

[2] Delavaran H, Jönsson A-C, Lövkvist H, Iwarsson S, Elmståhl S, Norrving B (2016). Cognitive function in stroke survivors: a 10-year follow-up study. Acta Neurol Scand.

[3] Schaapsmeerders P, Maaijwee NAM, Van Dijk EJ, Rutten-Jacobs LCA, Arntz RM, Schoonderwaldt HC (2013). Long-term cognitive impairment after first-ever ischemic stroke in young adults. Stroke.

[4] Ferreira MGR, Moro CHC, Franco SC (2015). Cognitive performance after ischaemic stroke. Dement Neuropsychol.

[5] Barker-Collo S, Starkey N, Lawes CMM, Feigin V, Senior H, Parag V (2012). Neuropsychological profiles of 5-year ischemic stroke survivors by oxfordshire stroke classification and hemisphere of lesion. Stroke.

[6] Nys GMS, Van Zandvoort MJE, De Kort PLM, Jansen BPW, Kappelle LJ, De Haan EHF (2005). Restrictions of the Mini-Mental State Examination in acute stroke. Arch Clin Neuropsychol.

[7] Nøkleby K, Boland E, Bergersen H, Schanke A-K, Farner L, Wagle J (2008). Screening for cognitive deficits after stroke: a comparison of three screening tools. Clin Rehabil.

[8] Lees R, Fearon P, Harrison JK, Broomfield NM, Quinn TJ (2012). Cognitive and mood assessment in stroke research. Stroke.

[9] Demeyere N, Riddoch MJ, Slavkova ED, Bickerton W-L, Humphreys GW (2015). The Oxford Cognitive Screen (OCS): validation of a stroke-specific short cognitive screening tool. Psychol Assess.

[10] Malloy PF, Cummings JL, Coffey CE, Duffy J, Fink M, Lauterbach EC (1997). Cognitive screening instruments in neuropsychiatry:a report of the Committee on Research of the American Neuropsychiatric Association. J Neuropsychiatry Clin Neurosci.

[11] Wilson JMG, Jungner G, World Health Organization (1968). Principles and practice of screening for disease/ JMG Wilson, G Jungner. Public Health Pap.

[12] Chan E, Khan S, Oliver R, Gill SK, Werring DJ, Cipolotti L (2014). Underestimation of cognitive impairments by the Montreal Cognitive Assessment (MoCA) in an acute stroke unit population. J Neurol Sci.

[13] Morris K, Hacker V, Lincoln NB (2012). The validity of the Addenbrooke's Cognitive Examination-Revised (ACE-R) in acute stroke. Disabil Rehabil.

[14] Pendlebury ST, Mariz J, Bull L, Mehta Z, Rothwell PM (2012). MoCA, ACE-R, and MMSE versus the National Institute of Neurological Disorders and Stroke-canadian Stroke Network vascular cognitive impairment harmonization standards neuropsychological battery after TIA and stroke. Stroke.

[15] Hachinski V, Iadecola C, Petersen RC, Breteler MM, Nyenhuis DL, Black SE (2006). National Institute of Neurological Disorders and Stroke-Canadian Stroke Network vascular cognitive impairment harmonization standards. Stroke.

[16] Lin HF, Chern CM, Chen HM, Yeh YC, Yao SC, Huang MF (2016). Validation of NINDS-VCI neuropsychology protocols for vascular cognitive impairment in Taiwan. PLoS One.

[17] Wong A, Xiong YY, Wang D, Lin S, Chu WWC, Kwan PWK (2013). The NINDS-Canadian stroke network vascular cognitive impairment neuropsychology protocols in Chinese. J Neurol Neurosurg Psychiatry.

[18] Dong Y, Xu J, Chan BP-L, Seet RCS, Venketasubramanian N, Teoh HL (2016). The Montreal cognitive assessment is superior to national institute of neurological disease and stroke-Canadian stroke network 5-minute protocol in predicting vascular cognitive impairment at 1 year. BMC Neurol.

[19] Chen X, Fan X, Zhao L, Duan L, Wang Z, Han Y (2015). Telephone-based cognitive screening for stroke patients in china. Int Psychogeriatr.

[20] Freitas S, Simões MR, Alves L, Vicente M, Santana I (2012). Montreal Cognitive Assessment (MoCA): validation study for vascular dementia. J Int Neuropsychol Soc.

[21] American Educational Research Association, American Psychological Association, National Council on Measurement in Education (2014). Standards for educational and psychological testing.

[22] Stolwyk RJ, O'Neill MH, McKay AJD, Wong DK (2014). Are cognitive screening tools sensitive and specific enough for use after stroke?: a systematic literature review. Stroke.

[23] Burton L, Tyson SF (2015). Screening for cognitive impairment after stroke: a systematic review of psychometric properties and clinical utility. J Rehabil Med.

[24] Cumming TB, Churilov L, Linden T, Bernhardt J (2013). Montreal Cognitive Assessment and Mini-mental State Examination are both valid cognitive tools in stroke. Acta Neurol Scand.

[25] Wong GKC, Lam SW, Wong A, Lai M, Siu D, Poon WS (2014). MoCA-assessed cognitive function and excellent outcome after aneurysmal subarachnoid hemorrhage at 1 year. Eur J Neurol.

[26] Wong GKC, Lam SW, Wong A, Mok V, Siu D, Ngai K (2014). Early MoCA-assessed cognitive impairment after aneurysmal subarachnoid hemorrhage and relationship to 1-year functional outcome. Transl Stroke Res.

[27] Boosman H, Visser-Meily JMA, Post MWM, Duits A, van Heugten CM (2013). Validity of the Barrow Neurological Institute (BNI ) screen for higher cerebral functions in stroke patients with good functional outcome. Clin Neuropsychol.

[28] Redfors P, Hofgren C, Eriksson I, Holmegaard L, Samuelsson H, Jood K (2014). The Barrow Neurological Institute screen for higher cerebral functions in cognitive screening after stroke. J Stroke Cerebrovasc Dis.

[29] Pan X, Chen H, Bickerton WL, Lau JKL, Kong APH, Rotshtein P (2015). Preliminary findings on the reliability and validity of the cantonese Birmingham Cognitive Screen in patients with acute ischemic stroke. Neuropsychiatr Dis Treat.

[30] Bickerton W-L, Demeyere N, Francis D, Kumar V, Remoundou M, Balani A (2015). The BCoS cognitive profile screen: utility and predictive value for stroke. Neuropsychology.

[31] Brookes RL, Hollocks MJ, Khan U, Morris RG, Markus HS (2015). The Brief Memory and Executive Test (BMET) for detecting vascular cognitive impairment in small vessel disease: a validation study. BMC Med.

[32] Lunardelli A, Mengotti P, Pesavento V, Sverzut A, Zadini A (2009). The Brief Neuropsychological Screening (BNS): valuation of its clinical validity. Eur J Phys Rehabil Med.

[33] Yoo DH, Lee JS (2016). Clinical usefulness of the clock drawing test applying rasch analysis in predicting of cognitive impairment. J Phys Ther Sci.

[34] Lees R, Selvarajah J, Fenton C, Pendlebury ST, Langhorne P, Stott DJ (2014). Test accuracy of cognitive screening tests for diagnosis of dementia and multidomain cognitive impairment in stroke. Stroke.

[35] Barnay J-L, Wauquiez G (2014). Feasibility of the Cognitive Assessment scale for Stroke Patients (CASP) vs. MMSE and MoCA in aphasic left hemispheric stroke patients. Ann Phys Rehabil Med.

[36] Benaim C, Barnay JL, Wauquiez G, Bonnin-Koang HY, Anquetil C, Pérennou D (2015). The Cognitive Assessment scale for Stroke Patients (CASP) vs. MMSE and MoCA in non-aphasic hemispheric stroke patients. Ann Phys Rehabil Med.

[37] Te Winkel-Witlox ACM, Post MWM, Visser-Meily JMA, Lindeman E (2008). Efficient screening of cognitive dysfunction in stroke patients: comparison between the CAMCOG and the R-CAMCOG, Mini Mental State Examination and Functional Independence Measure-cognition score. Disabil Rehabil.

[38] Cartoni A, Lincoln NB (2005). The sensitivity and specificity of the Middlesex Elderly Assessment of Mental State (MEAMS) for detecting cognitive impairment after stroke. Neuropsychol Rehabil.

[39] Oh HS, Kim JS, Shim EB, Seo WS (2015). Development and clinical validity of a mild vascular cognitive impairment assessment tool for Korean stroke patients. Asian Nurs Res (Korean Soc Nurs Sci).

[40] Pendlebury ST, Cuthbertson FC, Welch SJ V, Mehta Z, Rothwell PM (2010). Underestimation of cognitive impairment by Mini-Mental State Examination versus the Montreal Cognitive Assessment in patients with transient ischemic attack and stroke: a population-based study. Stroke.

[41] Zhou A, Jia J (2009). A screen for cognitive assessments for patients with vascular cognitive impairment no dementia. Int J Geriatr Psychiatry.

[42] Dong Y, Venketasubramanian N, Chan BP-L, Sharma VK, Slavin MJ, Collinson SL (2012). Brief screening tests during acute admission in patients with mild stroke are predictive of vascular cognitive impairment 3-6 months after stroke. J Neurol Neurosurg Psychiatry.

[43] Dong YH, Slavin MJ, Chan BPL, Venketasubramanian N, Sharma VK, Collinson SL (2014). Improving screening for vascular cognitive impairment at three to six months after mild ischemic stroke and transient ischemic attack. Int Psychogeriatrics.

[44] Dong Y, Slavin MJ, Chan BP-L, Venketasubramanian N, Sharma VK, Crawford JD (2013). Cognitive screening improves the predictive value of stroke severity scores for functional outcome 3-6 months after mild stroke and transient ischaemic attack: an observational study. BMJ Open.

[45] Xu Q, Cao WW, Mi JH, Yu L, Lin Y, Li YS (2014). Brief screening for mild cognitive impairment in subcortical ischemic vascular disease: a comparison study of the Montreal Cognitive Assessment with the Mini-mental State Examination. Eur Neurol.

[46] Shen YJ, Wang WA, Huang FD, Chen J, Liu HY, Xia YL (2016). The use of MMSE and MoCA in patients with acute ischemic stroke in clinical. Int J Neurosci.

[47] Van Der Wijst E, Wright J, Steultjens E (2014). The suitability of the Montreal Cognitive Assessment as a screening tool to identify people with dysfunction in occupational performance after mild stroke. Br J Occup Ther.

[48] Godefroy O, Fickl A, Roussel M, Auribault C, Bugnicourt JM, Lamy C (2011). Is the Montreal Cognitive Assessment superior to the Mini-mental State Examination to detect poststroke cognitive impairment?: A study with neuropsychological evaluation. Stroke.

[49] Salvadori E, Pasi M, Poggesi A, Chiti G, Inzitari D, Pantoni L (2013). Predictive value of MoCA in the acute phase of stroke on the diagnosis of mid-term cognitive impairment. J Neurol.

[50] Geubbels HJB, Nusselein BAM, Van Heugten CM, Valentijn SAM, Rasquin SMC (2015). Can the Montreal Cognitive Assessment predict discharge destination in a stroke population in the hospital?. J Stroke Cerebrovasc Dis.

[51] Campbell N, Rice D, Friedman L, Speechley M, Teasell RW (2016). Screening and facilitating further assessment for cognitive impairment after stroke: application of a shortened Montreal Cognitive Assessment (miniMoCA). Disabil Rehabil.

[52] Pendlebury ST, Welch SJ V, Cuthbertson FC, Mariz J, Mehta Z, Rothwell PM (2013). Telephone assessment of cognition after transient ischemic attack and stroke: modified telephone interview of cognitive status and telephone Montreal Cognitive Assessment versus face-to-face Montreal Cognitive Assessment and neuropsychological battery. Stroke.

[53] Sahathevan R, Ali KM, Ellery F, Mohamad NF, Hamdan N, Ibrahim NM (2014). A Bahasa Malaysia version of the Montreal Cognitive Assessment: validation in stroke. Int Psychogeriatr.

[54] Zuo L, Dong Y, Zhu R, Jin Z, Li Z, Wang Y (2016). Screening for cognitive impairment with the Montreal Cognitive Assessment in Chinese patients with acute mild stroke and transient ischaemic attack: a validation study. BMJ Open.

[55] Tu Q-Y, Jin H, Ding B-R, Yang X, Lei Z-H, Bai S (2013). Reliability, validity, and optimal cutoff score of the Montreal Cognitive Assessment (Changsha version) in ischemic cerebrovascular disease patients of hunan province, China. Dement Geriatr Cogn Dis Extra.

[56] You J-S, Chen R-Z, Zhang F-M, Zhou Z-Y, Cai Y-F, Li G-F (2011). The chinese (cantonese) montreal cognitive assessment in patients with subcortical ischemic vascular dementia. Dement Geriatr Cogn Dis Extra.

[57] Wong GKC, Lam SW, Wong A, Ngai K, Poon WS, Mok V (2013). Comparison of Montreal Cognitive Assessment and Mini-Mental State Examination in evaluating cognitive domain deficit following aneurysmal subarachnoid haemorrhage. PLoS One.

[58] Wu Y, Wang M, Ren M, Xu W (2013). The effects of educational background on Montreal Cognitive Assessment screening for vascular cognitive impairment, no dementia, caused by ischemic stroke. J Clin Neurosci.

[59] Wong GKC, Lam S, Ngai K, Wong A, Mok V, Poon WS (2012). Evaluation of cognitive impairment by the Montreal Cognitive Assessment in patients with aneurysmal subarachnoid haemorrhage: prevalence, risk factors and correlations with 3 month outcomes. J Neurol Neurosurg Psychiatry.

[60] Wong A, Nyenhuis D, Black SE, Law LSN, Lo ESK, Kwan PWL (2015). Montreal Cognitive Assessment 5-Minute protocol is a brief, valid, reliable, and feasible cognitive screen for telephone administration. Stroke.

[61] Wong A, Xiong YY, Kwan PWL, Chan AYY, Lam WWM, Wang K (2009). The validity, reliability and clinical utility of the Hong Kong Montreal Cognitive Assessment (HK-MoCA) in patients with cerebral small vessel disease. Dement Geriatr Cogn Disord.

[62] Dong Y, Sharma VK, Chan BPL, Venketasubramanian N, Teoh HL, Seet RCS (2010). The Montreal Cognitive Assessment (MoCA) is superior to the Mini-Mental State Examination (MMSE) for the detection of vascular cognitive impairment after acute stroke. J Neurol Sci.

[63] Williams PM, Johnson C, Swan S, Barber C, Murphy P, Devine J (2016). The Northwick Park Examination of Cognition: a brief cognitive assessment tool for use in acute stroke services. Int J Ther Rehabil.

[64] Demeyere N, Riddoch MJ, Slavkova ED, Jones K, Reckless I, Mathieson P (2016). Domain-specific versus generalized cognitive screening in acute stroke. J Neurol.

[65] Larson E, Kirschner K, Bode R, Heinemann A, Goodman R (2005). Construct and predictive validity of the repeatable battery for the assessment of neuropsychological status in the evaluation of stroke patients. J Clin Exp Neuropsychol.

[66] Hobson P, Meara J, Taylor C (2007). The Weigl Colour-Form Sorting Test: A quick and easily administered bedside screen for dementia and executive dysfunction. Int J Geriatr Psychiatry.

[67] Tobler-Ammann BC, de Bruin ED, Brugger P, de Bie RA, Knols RH (2016). The Zürich Maxi Mental Status Inventory (ZüMAX): test-retest reliability and discriminant validity in stroke survivors. Cogn Behav Neurol.

[68] Walter SD, Eliasziw M, Donner A (1998). Sample size and optimal designs for reliability studies. Stat Med.

[69] Lees R, Lua J, Melling E, Miao Y, Tan J, Quinn TJ (2014). Cog-4 has limited diagnostic test accuracy and validity for cognitive assessment in stroke survivors. J Stroke Cerebrovasc Dis.

[70] Blake H, McKinney M, Treece K, Lee E, Lincoln NB (2002). An evaluation of screening measures for cognitive impairment after stroke. Age Ageing.

